# Divergent domains of *28S* ribosomal RNA gene: DNA barcodes for molecular classification and identification of mites

**DOI:** 10.1186/s13071-020-04124-z

**Published:** 2020-05-13

**Authors:** Yae Zhao, Wan-Yu Zhang, Rui-Ling Wang, Dong-Ling Niu

**Affiliations:** 1 Department of Pathogen Biology and Immunology, School of Basic Medical Sciences, Xi’an Jiaotong University, Xi’an, 710061 Shaanxi People’s Republic of China; 2 Assisted Reproduction Center, Northwest Women’s and Children’s Hospital, Xi’an, 710003 Shaanxi People’s Republic of China

**Keywords:** Mites, rDNA 28S, Universal primers, Divergent regions, Molecular identification, DNA barcode

## Abstract

**Background:**

The morphological and molecular identification of mites is challenging due to the large number of species, the microscopic size of the organisms, diverse phenotypes of the same species, similar morphology of different species and a shortage of molecular data.

**Methods:**

Nine medically important mite species belonging to six families, i.e. *Demodex folliculorum*, *D. brevis*, *D. canis*, *D. caprae*, *Sarcoptes scabiei canis*, *Psoroptes cuniculi*, *Dermatophagoides farinae*, *Cheyletus malaccensis* and *Ornithonyssus bacoti*, were collected and subjected to DNA barcoding. Sequences of *cox*1, *16S* and *12S* mtDNA, as well as ITS, *18S* and *28S* rDNA from mites were retrieved from GenBank and used as candidate genes. Sequence alignment and analysis identified *28S* rDNA as the suitable target gene. Subsequently, universal primers of divergent domains were designed for molecular identification of 125 mite samples. Finally, the universality of the divergent domains with high identification efficiency was evaluated in Acari to screen DNA barcodes for mites.

**Results:**

Domains D5 (67.65%), D6 (62.71%) and D8 (77.59%) of the *28S* rRNA gene had a significantly higher sequencing success rate, compared to domains D2 (19.20%), D3 (20.00%) and D7 (15.12%). The successful divergent domains all matched the closely-related species in GenBank with an identity of 74–100% and a coverage rate of 92–100%. Phylogenetic analysis also supported this result. Moreover, the three divergent domains had their own advantages. D5 had the lowest intraspecies divergence (0–1.26%), D6 had the maximum barcoding gap (10.54%) and the shortest sequence length (192–241 bp), and D8 had the longest indels (241 bp). Further universality analysis showed that the primers of the three divergent domains were suitable for identification across 225 species of 40 families in Acari.

**Conclusions:**

This study confirmed that domains D5, D6 and D8 of *28S* rDNA are universal DNA barcodes for molecular classification and identification of mites. *28S* rDNA, as a powerful supplement for *cox*1 mtDNA 5’-end 648-bp fragment, recommended by the International Barcode of Life (IBOL), will provide great potential in molecular identification of mites in future studies because of its universality.
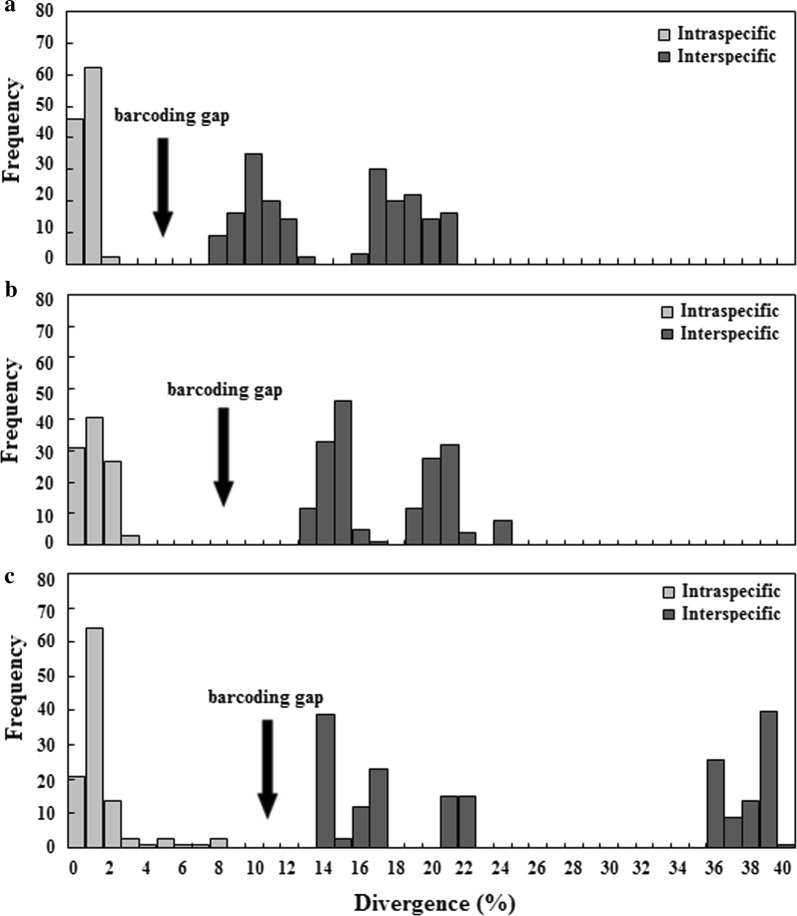

## Background

Mites are a large group of microscopic arthropods, which exist widely in the natural environment, and are classified as Arachnida, Acari, Acariformes and Parasitiformes. For a long time, the classification of mites has mainly been dependent on morphological characteristics and/or parasitic hosts. However, this field has been facing challenges due to the large number of species, the microscopic size of the organisms, diverse phenotypes of the same species, similar morphology of different species, and a shortage of specialized taxonomists [1–3]. Medically important mites can serve as pathogens, allergens, or microorganism reservoirs and are significant for human and animal health. *Sarcoptes*, *Demodex* or *Psoroptes* mites as pathogens can directly infect humans or mammals and cause skin or external auditory canal lesions [4–7]. *Dermatophagoides farinae*, *D. pteronyssinus* and *Euroglyphus maynei* as allergen mites can cause allergic diseases [8]. Some species of gamasid mites and chigger mites, as vectors or reservoirs, can result in the spread of hemorrhagic fever with renal syndrome, scrub typhus and other acute infectious diseases [9, 10]. Therefore, in order to take targeted measures to control mite-borne diseases, it is extremely important to distinguish medically important mites effectively.

Although rapidly developed molecular biology techniques have provided support for classification and identification of mites [11–14], there are still some problems with the classification technique of DNA barcoding: (i) a shortage of molecular data. More than 50,000 mite species have been identified in nature [15], whereas less than 100 species have molecular data available on GenBank. Therefore, for most species the gene sequence template for designing primers is not available, and thus they cannot be identified by molecular techniques; (ii) the limited research reports. Only a small number of mite species have been identified by DNA barcoding, mainly based on *cox*1 [16–26], *16S* and *12S* mtDNA gene fragments [2, 27–32], especially *cox*1 648-bp fragment. However, these studies usually involve one family, one genus, and a few species. In addition, there is a lack of clarity regarding the universality and identification efficiency of the primers as they are located at different positions of the same gene sequences in different species; and (iii) fewer reports on rDNA barcodes compared to mtDNA. The limited reports on rDNA are mainly concentrated on the ITS region [15, 19, 21], for which primers are not universal as they are located in different positions of sequences which are lacking in some mite species. It is unusual for *18S* and *28S* rRNA genes to be used for molecular identification at lower taxonomic ranks (species and genus) [14, 18, 33–35], which may result from the traditional opinion that both are conserved and thus suitable for identification at the higher taxonomic ranks. However, their true value in species identification at lower taxonomic ranks might be underestimated.

In the present study, 125 mite samples of nine medically important mite species belonging to six families were collected and identified based on morphology. Subsequently, sequences of the *cox*1, *16S* and *12S* mtDNA regions, and the ITS, *18S* and *28S* rDNA regions, were downloaded from GenBank for sequence alignment, and previously reported primers used for molecular identification of the nine mite species were marked. Based on the universality analysis of these primers, *28S* rDNA, which is composed of alternated conserved regions and divergent regions with large sequence differences, was screened and confirmed as the target gene. Universal or degenerate primers of D2, D3, D5, D6, D7 and D8 regions were designed specifically for molecular identification of the nine mite species. By comparing the sequencing success rate, intraspecific divergence, and DNA barcoding gap of the six divergent regions, D5, D6 and D8 regions, were confirmed as DNA barcodes for the nine mite species. Finally, all partial *28S* rRNA gene sequences of species of the Acariformes and Parasitiformes were retrieved from GenBank and aligned to verify the universality of the primers of the DNA barcodes. In summary, this study aimed to provide a novel approach for molecular classification of mites, and thereby to solve the difficulty in morphological and molecular identification through screening universal regions to be used in DNA barcoding of medically important mites.

## Methods

### Collection and morphological classification of mites

Medically important mites collected in this study were classified morphologically by two approaches. First, some mites were directly classified according to their parasitic habitats and hosts, including *Ornithonyssus bacoti* from rat-lingering areas [10], *Psoroptes cuniculi* isolated from the external auditory canal of rabbits [36], *Sarcoptes scabiei canis* [26], *Demodex canis* [37] collected from dogs suffering from mange and demodicosis, and *D. caprae* isolated from skin nodules of goats [38]. Next, mites of different species residing in the same habitat or host were further identified according to morphological characteristics such as size, shape, color, dermatoglyph, and chelicerae. These included *D. farinae* [39] and *Cheyletus malaccensis* [40] breeding in a flour-processing workshop, and *D. folliculorum* and *D. brevis* collected from humans [1]. A total of 125 mite samples were collected. Images were taken using a light microscope (MOTIC, Xiamen, China).

### Target gene screening and universal primer design

The gene sequences of *cox*1, *16S* and *12S* mtDNA, and ITS, *18S* and *28S* rDNA for species of the six mite families involved in our study were downloaded from GenBank for alignment. The universality of the commonly used primers reported previously was analyzed based on the alignment results. Multiple pairs of universal or degenerate primers were designed in the conserved regions of the *28S* rDNA.

### Molecular identification of medically important mites

Genomic DNA was extracted from individual mites using the Chelex-100 method and used directly for PCR amplification and cloning as previously described in Cheng et al. [36]. Positive clones were sent to Genewiz Biological Co., Ltd (Suzhou, China) for sequencing. For each *28S* rRNA gene fragment, five sequences were obtained for most mites, fewer than 5 sequences were obtained for only a few mites. Each sequence was assigned to a species according to its coverage rate and identity with the corresponding sequence in the GenBank database by BLAST analysis. Based on the sequencing success rate, candidate DNA barcodes were preliminarily screened.

### Phylogenetic analysis

Phylogenetic trees were reconstructed using maximum likelihood (ML) and Bayesian inference (BI) methods. The mite sequences of candidate DNA barcodes obtained in this study and the closely related species deposited in GenBank were aligned using Clustal X 1.8 with the multiple alignment model. The ML trees were reconstructed using the Kimura 2-parameter (K2P) model in MEGA 5.0 [41], with each node supported by 1000 bootstraps. Before BI analysis, the most appropriate models were selected based on Akaikeʼs information criterion (AIC) in MrModeltest 2.3 and Modeltest 3.7 [42, 43]. In terms of fit and parsimony, the model with the lowest AIC value was identified as the best [44]. The GTR + I + G model was used for nucleotide sequences of *28S* rDNA D5 and D6, the HKY + I + G model was used for D8. The BI trees were performed in MrBayes 3.2.1 [45]. The Markov chain was run with 2,000,000 generations, and trees were sampled every 100th generation. The first 25% of samples were discarded as ‘burn-in’, and the remaining data were used to generate a 50% majority-consensus tree. The phylogenetic trees were visualized using TreeGraph 2 [46].

### Sequence divergence and DNA barcoding gap

Taking each family involved in this study as a unit, the intraspecific divergences and interspecific divergences of each *28S* rRNA gene fragment were calculated using MEGA 5.0. Frequency distribution plots of divergences were drawn using SPSS 18.0. According to the screening criteria for ideal DNA barcodes [2], DNA barcodes were confirmed for the nine mite species.

### Universality analysis

Using “Acari” and “*28S* rRNA” as keywords, the partial sequences over 3000 bp were downloaded from GenBank. One representative sequence of each species was chosen for alignment in Clustal X 1.8. Taking the almost complete *28S* rDNA sequence of *D. farinae* (GenBank: JQ000555) as a template, the universal primers designed for the screened DNA barcodes were marked in order to analyze their universality in the Acariformes and Parasitiformes.

## Results

### Collection and morphological classification of mites

**Fig. 1:**
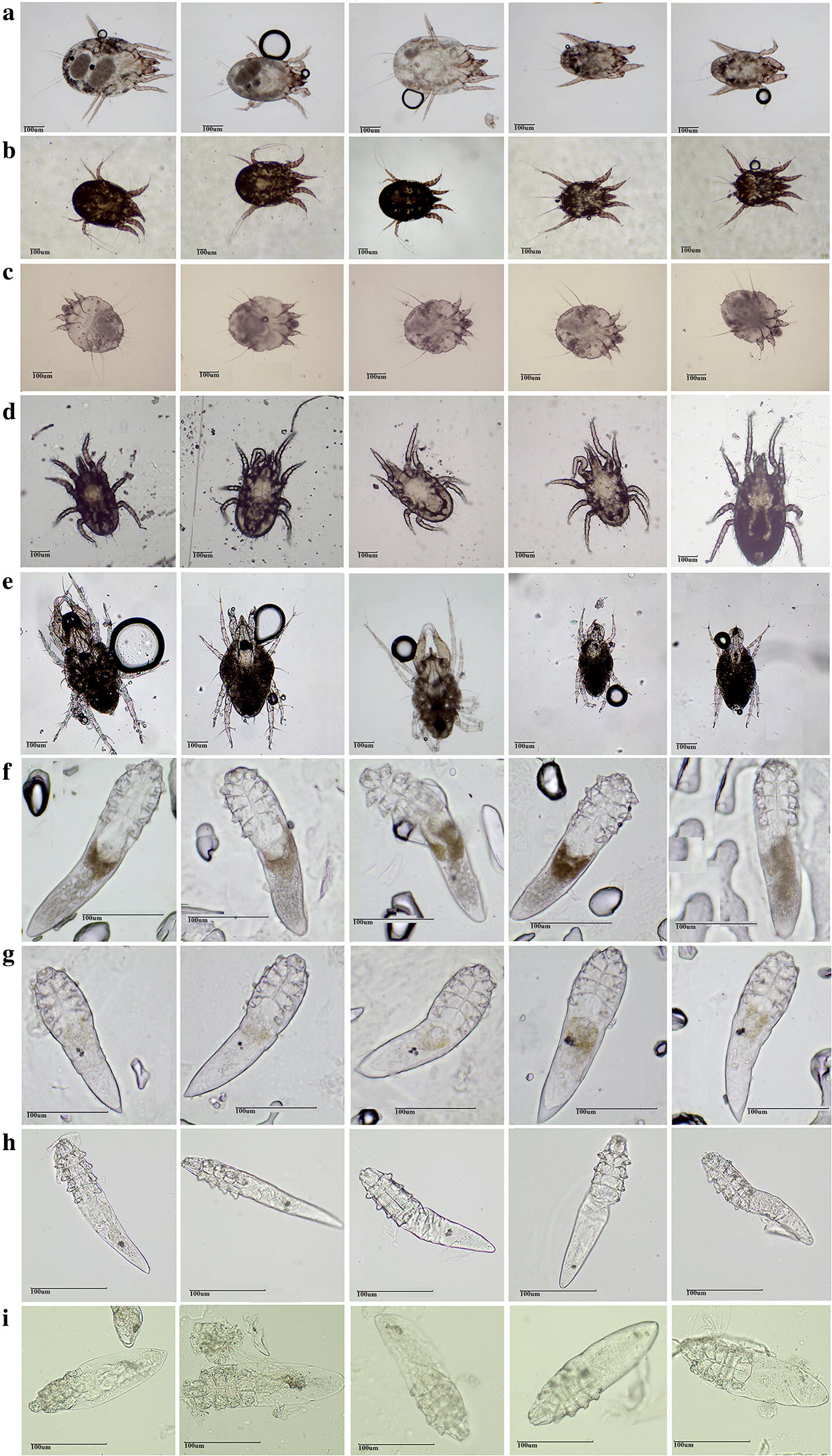
Adult morphology of the nine mite species. **a***Dermatophagoides farinae* (10 × 10); **b***Psoroptes cuniculi* (10 × 4); **c***Sarcoptes scabiei canis* (10 × 10); **d***Ornithonyssus bacoti* (10 × 10); **e***Cheyletus malaccensis* (10 × 10); **f***Demodex folliculorum* (10 × 40); **g***Demodex brevis* (10 × 40); **h***Demodex canis* (10 × 40); **i***Demodex caprae* (10 × 40). *Scale-bars*: 100 µm

A total of nine medically important mite species were obtained in this study, including *D. farinae* (family Pyroglyphidae), *P. cuniculi* (family Psoroptidae), *S. canis* (family Sarcoptidae), *O. bacoti* (family Macronyssidae), *C. malaccensis* (family Cheyletidae), and *D. folliculorum*, *D. brevis*, *D. canis* and *D. caprae* (all of the family Demodicidae). Five mites were obtained for *O. bacoti*, 10 mites each for *D. farinae*, *P. cuniculi*, *S. canis* and *C. malaccensis*, and 20 mites each for the four *Demodex* species (*D. folliculorum*, *D. brevis*, *D. canis* and *D. caprae*). A total of 125 mites were photographed and identified based on morphology, and then used for genomic DNA extraction (Fig. 1).

### Target gene screening and universal primer design

#### mtDNA gene fragments

Table 1 and Additional file 1: Figure S1 show the data for a total of 33 *cox*1 mtDNA gene sequences of the nine mite species retrieved from GenBank [2, 10, 36]. Taking the mtDNA complete sequence of *D. farinae* (GenBank: NC_013184) as a template, the sequence alignment showed that the forward primer of *S. canis*, and the forward and reverse primers of the *C. malaccensis* aligned well with the universal primers reported by Hebert et al. [47]. However, the other seven mite species had sequences of different lengths, and the primers were located at different positions where mutations or deletions occurred. Therefore, the *cox*1 mtDNA gene did not satisfy the requirements for designing universal primers.

**Table 1:** Reported gene primers for medically important mites

Mite	Gene	Length (bp)	Primers (5’-3’)	Reference
*D. farinae*	*cox*1	832 (4–835)	F: GGACGATGATTAATATCCAC	Zhao et al. unpublished
			R: CAATAGACACTATAGCATAAATCA	
	*16S*	402 (65–466)	F: TCACACTAAGGTAGCGAG	
			R: ACGCCGACTTTAACTCA	
	*12S*	837 (61–897)	F: CGGCTTTGGGAGTTGTAGAA	
			R: CTTATCTATCAAAAGAGTGACGGG	
	ITS2	322 (116–437)	F: CGAACGCATATTGCAGCCATT	
			R: ATTCAGGGGGTAATCTCGCTTG	
*P. cuniculi*	*cox*1	652 (61–712)	F: GGTGTGTGAAGTGGTATATTG	Cheng et al. [36]
			R: GCCCAAAAAACCAGAAA	
	ITS2	275 (108–382)	F: GGCTTCGTTTGTCTGAG	
			R: GTAATCTCGCTTGATCTGA	
*S. canis*	*cox*1	819 (27–845)	F: GGTCAACAAATCATAAAGA	Zhao et al. [26]
			R: CAATAGAAATCATAGCATAAA	
	*16S*	320 (94–413)	F: TAGGGTCTTTTTGTCTTG	
			R: TATTATAAAAATATTAAATTAAAAAATTAG	
	*12S*	743(61–803)	F: CATTAAGCAATTTCCCCA	
			R: CTAAACAAGAAACACTTTCCA	
	ITS2	417 (94–510)	F: AGTATCCGATGGCTTCGTTTGTCT	
			R: AATCCCGTTTGGTTTCTTTTCCTC	
*O. bacoti*	*cox*1	479 (442–920)	F: CTTCATATYGCWGGTATTTCTT	Zhao et al. [10]
			R: GTAGCTGTAGTAAAATARGCTCG	
	*16S*	267 (97–363)	F: TGCTAAGAGAATGGAWDAA	
			R: CATCGRGGTCGCAAACT	
	ITS	510 (1–534)	F: GGTTTCCGTAGGTGAACC	
			R: CACTTGATTTCAGATACACGT	
*C. malaccensis*	*cox*1	709 (27–735)	F: GGTCAACAAATCATAAAGATATTG	Zhao et al. unpublished
			R: TAAACTTCAGGGTGACCAAAA	
	*12S*	388 (445–832)	F: CTAGGATTAGATACCCTATTA	
			R: ACTATGTTACGACTTATCTATC	
*Demodex* mites	*cox*1	750 (64–813)	F: CACATAGAACTTTCAATTCTAAA	Hu et al. [2]
			R: CTCCTGCTGGGTCGAA	
	*16S*	337 (52–388)	F: GAGGTATTTTGACTGTGCTA	
			R: TCAAAAGCCAACATCG	
	*12S*	757 (79–835)	F: CTACTTTGTTACGACTTATTTTA	
			R: GCCAGCAGTTTCGGTTA	

*Abbreviations*: F, forward; R, reverse; Tm, melting temperature

A total of 25 *16S* mtDNA gene sequences were downloaded; no sequence was available for *C. malaccensis*. The sequence alignment showed that the primer positions of *Demodex* mites, *D. farinae* and *O. bacoti* were close, with the forward primers located at 570–610 bp and the reverse primers located at 900–1000 bp. However, the sequences were not conserved, and therefore, universal primers could not be designed.

A total of 22 *12S* mtDNA gene sequences were downloaded; no sequence was available for *O. bacoti*. For *S. canis*, *D. farinae*, and four *Demodex* mites, the forward primers were located at 190–230 bp, and the reverse primers were located at 790–840 bp. Nevertheless, the primer sequences differed greatly, so once again, universal primers could not be designed.

As the reported primers of *cox*1, *16S* and *12S* mtDNA genes were not universal for the nine mite species, these three genes could not be considered as the target genes for molecular identification in this study.

#### rRNA gene fragments

A total of 12 ITS rRNA gene sequences were obtained from GenBank; no sequences were available for species of the Cheyletidae and Demodicidae. As ITS2 sequences were too variable to be aligned, universal primers could not be designed (Additional file 2: Figure S2a). Therefore, the ITS rRNA was considered to be an unsuitable DNA barcode candidate.

A total of 18 complete *18S* RNA gene sequences were obtained; no sequences were available for *O. bacoti* and *S. canis*. The sequence alignment showed that the *18S* rRNA gene sequences were conserved and suitable for universal primer design; however, the sequence divergences were too small to identify the species efficiently. Thus, *18S* rRNA was also not considered a suitable DNA barcode candidate gene (Additional file 2: Figure S2b).

Only 7 almost complete *28S* rRNA gene sequences were obtained with sequences lacking for *D. canis* and *D. caprae*. Sequence alignment showed that *28S* rDNA was significantly more variable than *18S* rDNA, and obvious conserved regions were observed among D2, D3, D4, D5, D6, D7 and D8 regions (Additional file 3: Figure S3). Therefore, universal primers were successfully designed in the present study, with degenerate bases used for specific mutation sites (Table 2).

**Table 2:** Universal or degenerate primers for divergent regions of *28S* rDNA for the nine mite species

Gene fragment	Length (bp)	Primers (5’-3’)	Tm (°C)
D2	532	F: AACAAGTAC**H**GTGA**K**GGAA	52
		R: CTCCTTGGTCCGTGTTTC	
D3	252	F: GTCTTGAAACACGGACCAA	54
		R: ATCGATTTGCACGTCAGAA	
D5	419	F: TTCTGACGTGCAAATCGAT	55
		R: GGCAGGTGAGTTGTTACACA	
D6	202	F: GTGTGTAACAACTCACCTGCC	55
		R: TTGCTACTACCACCAAGATCTG	
D7	465	F: CAGATCTTGGTGGTAGTAGCA	55
		R: CCTTGGAGACCTGCTGC	
D8	314	F: GCA**K**CAGGTCTCCAAGG	52
		R: GTTTTAATTAGACAGTCGGATTC	

*Abbreviations*: F, forward; R, reverse; Tm, melting temperature

*Note*: The letters in bold in primers indicate degenerate bases

### Molecular identification of nine mite species using divergent regions of *28S* rDNA

#### PCR amplification, sequencing and alignment

The sequencing results and BLAST analysis of the nine mite species (Table 3) showed that the sequencing success rates for D2 (19.20%, 24/125), D3 (20.00%, 25/125) and D7 regions (15.12%, 13/86) were low due to the lower success rates for the four *Demodex* species (3.18%, 7/220). However, the sequencing success rates for D5 (67.65%, 46/68), D6 (62.71%, 37/59), and D8 (77.59%, 45/58) were significantly higher. Additionally, using the latter regions, the nine mite species were all matched with the corresponding species with sequences available on GenBank, with coverage rates of 92–100% and identities of 74–100%. Therefore, we concluded that D5, D6 and D8 regions could be considered as candidate DNA barcodes, while D2, D3 and D7 regions were excluded from further analysis.

**Table 3:** Sequencing results of the six divergent regions of *28S* rDNA for the nine mite species

DD	Mite species	Length	SR%	GenBank ID	CRS	GenBank ID	I/C
D2	*D. farinae*	450–542	20.0 (2/10)	MH540331–32	*D. farinae*	JQ000555	98/100
	*P. cuniculi*	533	40.0 (4/10)	MH540338–41	*P. ovis*	JQ000549	98/100
	*S. canis*	465–550	10.0 (1/10)	MH540342	*Mesalgoides* sp.	JQ000537	77/100
	*O. bacoti*	465	100 (5/5)	MH540333–37	*O. bursa*	FJ911789	92/100
	*C. malaccensis*	617	10.0 (1/10)	MH540329	*H. simplex*	KP325024	99/100
	*D. folliculorum*	/	0 (0/20)	–	–	–	–
	*D. brevis*	/	0 (0/20)	–	–	–	–
	*D. canis*	/	0 (0/20)	–	–	–	–
	*D. caprae*	773	5.0 (1/20)	MH540330	*H. holopus*	KY922056	74/34
D3	*D. farinae*	252	50.0 (5/10)	MH001311–15	*D. farinae*	JQ000555	99/100
	*P. cuniculi*	252–256	40.0 (4/10)	MH001324–27	*P. ovis*	JQ000549	99/100
	*S. canis*	256	0 (0/10)		–	–	–
	*O. bacoti*	256	100 (5/5)	MH001318–22	*C. haematophagus*	FJ911790	99/100
	*C. malaccensis*	312–313	50.0 (5/10)	MH001306–10	*Oudemansicheyla* sp.	KP276422	76/100
	*D. folliculorum*	–	0 (0/20)	–	–	–	–
	*D. brevis*	358–362	20.0 (4/20)	MH540325–28	*D. folliculorum*	KY922058	78/96
	*D. canis*	368	10.0 (2/20)	MH540323–24	*D. folliculorum*	KY922058	86/95
	*D. caprae*	–	0 (0/20)	–	–	–	–
D5	*D. farinae*	419	50.0 (5/10)	MH001255–59	*D. farinae*	JQ000555	100/100
	*P. cuniculi*	419	60.0 (6/10)	MH001275–80	*P. ovis*	JQ000549	100/100
	*S. canis*	413–423	30.0 (3/10)	MH001267–69	*Cystoidosoma* sp.	JQ000474	97/100
	*O. bacoti*	413	100 (5/5)	MH001260–64	Melicharidae sp.	KP276394	99/100
	*C. malaccensis*	436	100 (5/5)	MH001270–74	*Oudemansicheyla* sp.	KP276422	93/100
	*D. folliculorum*	456	83.3 (5/6)	KY305302–05, KY347877	*D. folliculorum*	KY347877	99/100
	*D. brevis*	438–442	75.0 (6/8)	KY305306–11	*D. brevis*	HQ718592	98/100
	*D. canis*	445–459	85.7 (6/7)	KY305312–17	*D. folliculorum*	KY347877	93/100
	*D. caprae*	502–507	71.4 (5/7)	KY305318–22	*D. folliculorum*	KY347877	83/100
D6	*D. farinae*	202	40.0 (2/5)	MH001286, MH001290	*D. farinae*	JQ000555	99/100
	*P. cuniculi*	192–200	40.0 (2/5)	MH001296, MH001300	*P. ovis*	JQ000549	99/100
	*S. canis*	195–202	20.0 (1/5)	MH001301	*C. amazonae*	KP325029	93/100
	*O. bacoti*	195	83.3 (5/6)	MH001291–95	Melicharidae sp.	KP276394	98/100
	*C. malaccensis*	195–198	40.0 (4/10)	MH001281–84	*Oudemansicheyla* sp.	KP276422	93/100
	*D. folliculorum*	241	100 (6/6)	KY305302–05, KY347876–77	*D. folliculorum*	KY347876	99/100
	*D. brevis*	232	75.0 (6/8)	KY305306–11	*D. brevis*	HQ718592	99/100
	*D. canis*	201	85.7 (6/7)	KY305312–17	*D. brevis*	HQ718592	85/100
	*D. caprae*	228–230	71.4 (5/7)	KY305318–22	*D. brevis*	HQ718592	90/100
D7	*D. farinae*	450–460	33.3 (2/6)	MH001334,36	*D. farinae*	JQ000555	100/100
	*P. cuniculi*	450–468	30.0 (3/10)	MH001343, MH001346, MH001347	*P. ovis*	JQ000549	100/100
	*S. canis*	450–501	30.0 (3/10)	MH001349–51	*A. ocelatus*	JQ000623	89/100
	*O. bacoti*	450	50.0 (5/10)	MH001338–42	Laelapidae sp.	KP276392	96/100
	*C. malaccensis*	450	0/15	–	–	–	–
	*D. folliculorum*	450	0/15	–	–	–	–
	*D. brevis*	450	0/15	–	–	–	–
	*D. canis*	450	0/15	–	–	–	–
	*D. caprae*	450	0/15	–	–	–	–
D8	*D. farinae*	314	100 (5/5)	MH001240–44	*D. farinae*	JQ000555	100/100
	*P. cuniculi*	314	100 (5/5)	MH001250–54	*P. ovis*	JQ000549	100/100
	*S. canis*	302–322	30.0 (3/10)	MH001229, MH001230, MH001234	*C. amazonae*	KP325029	87/100
	*O. bacoti*	302	100 (5/5)	MH001245–49	Laelapidae sp.	KP276392	95/100
	*C. malaccensis*	393	50.0 (5/10)	MH001235–39	*Oudemansicheyla* sp.	KP276422	74/100
	*D. folliculorum*	519	100 (5/5)	KY305323–27	*D. folliculorum*	HQ728004	100/100
	*D. brevis*	428–432	85.7 (6/7)	KY305328–33	*D. folliculorum*	HQ728004	96/92
	*D. canis*	541–543	100 (5/5)	KY305334–38	*D. canis*	HQ728002	99/100
	*D. caprae*	516	100 (6/6)	KY305339–44	*D. folliculorum*	HQ728004	86/100

*Abbreviations*: DD, divergent domains; SR, sequencing success rate; CRS, closely related species; I/C, identity/coverage; *D. farinae*, *Dermatophagoides farinae*; *P. cuniculi*, *Psoroptes cuniculi*; *S. canis*, *Sarcoptes scabiei canis*; *O. bacoti*, *Ornithonyssus bacoti*; *C. malaccensis*, *Cheyletus malaccensis*; *D. folliculorum*, *Demodex folliculorum*; *D. brevis*, *Demodex brevis*; *D. canis*, *Demodex canis*; *D. caprae*, *Demodex caprae*; *P. ovis*, *Psoroptes ovis*; *O. bursa*, *Ornithonyssus bursa*; *H. simplex*, *Haplochthonius simplex*; *H. holopus*, *Harpypalpus holopus*; *C. haematophagus*, *Chiroptonyssus haematophagus*; *C. amazonae*, *Chirnyssoides amazonae*; *A. ocelatus*, *Amerodectes aff. ocelatus*

#### Phylogenetic relationships

**Fig. 2:**
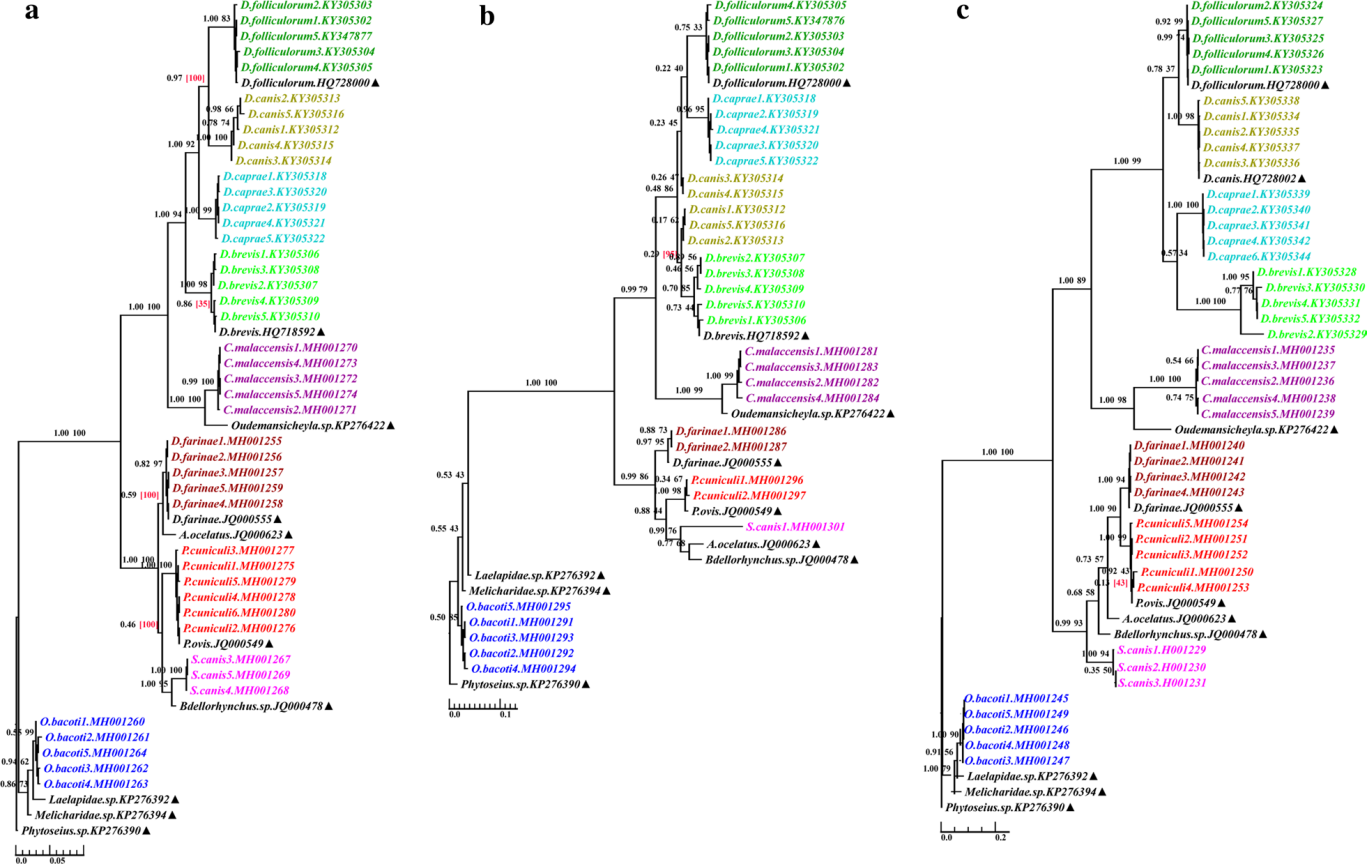
Molecular phylogeny of the nine mite species and the closely related species based on data for the three divergent regions of *28S* rDNA: D5 (**a**); D6 (**b**); D8 (**c**). Support values are shown above branches. The numbers in parentheses represent ML conflicting support values (which means the conflicting topology). *Phytoseius* sp. (GenBank: KP276390) was used as the outgroup. Triangles indicate sequences retrieved from GenBank

The ML and BI trees based on *28S* rDNA of D5, D6 and D8 yielded generally congruent topologies (Fig. 2). The bootstrap support (bs) and posterior probability (pp) values showed a similar trend, and posterior probability support was generally higher than bootstrap support, showing a more credible phylogenetic structure. The mites of the same species formed stable phylogenetic branches. The nine mite species formed distinct clusters within three major groups. The first group comprised the four *Demodex* species, *C. malaccensis* and the closely related species, all belonging to the Cheyletoidea in Acariformes (pp of 1.00, 0.99 and 1.00 in BI trees derived from D5, D6 and D8, respectively). The second group was formed by *S. canis*, *P. cuniculi*, *D. farinae* and the corresponding closely related species, all belonging to the Psoroptidia in Acariformes (pp of 1.00, 0.99 and 0.99 in BI trees derived from D5, D6 and D8, respectively). *Ornithonyssus bacoti* and its closely related species of Parasitiformes were clustered as a single group (pp of 1.00, 1.00 and 1.00 in BI trees derived from D5, D6 and D8, respectively). These clustering results were in accordance with the morphological identification.

#### Comparison of DNA barcode candidates

**Fig. 3:**
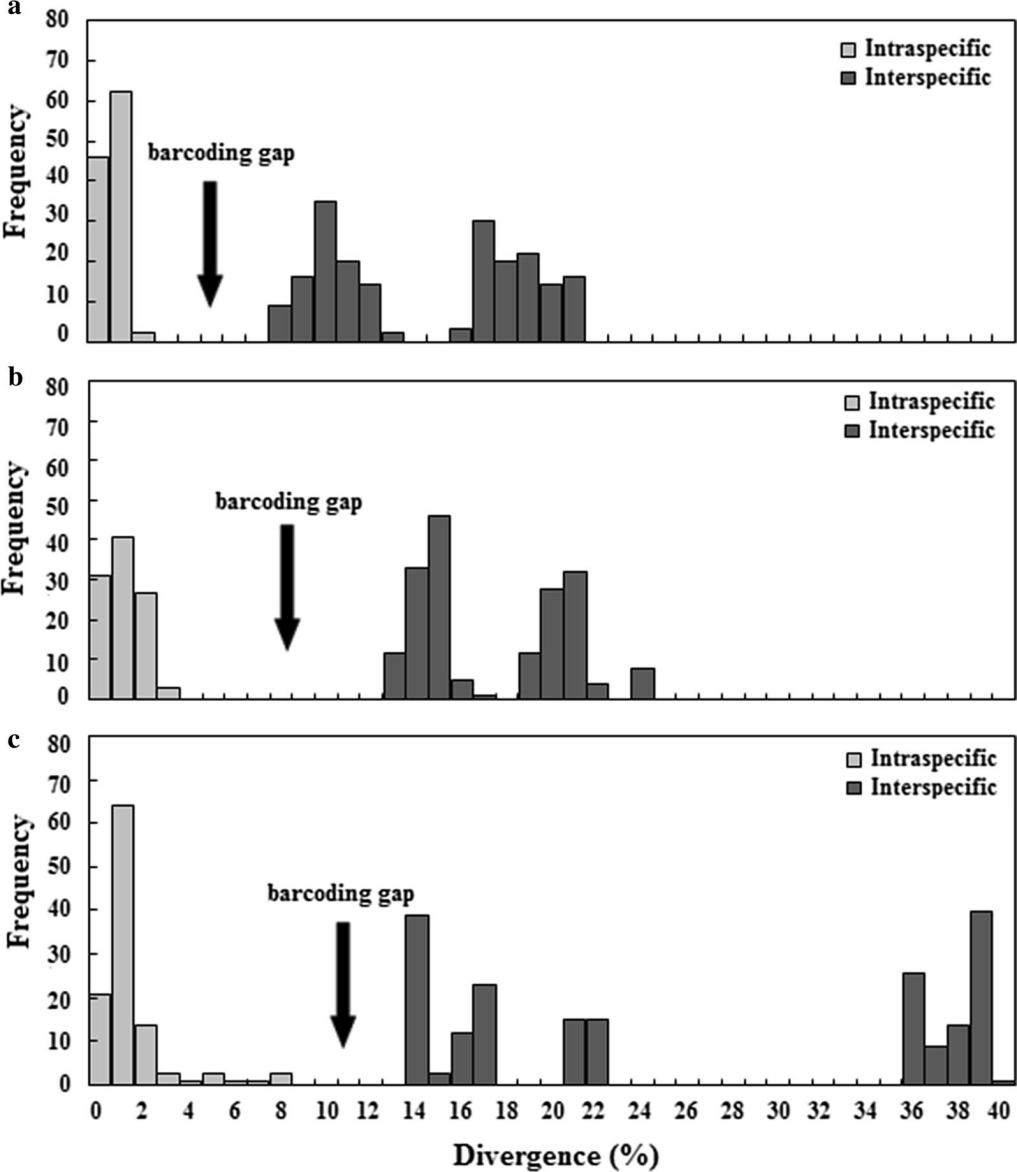
Frequency histogram plots of divergence for the three candidate DNA barcodes. **a** D5; **b** D6; **c** D8

Table 4 and Fig. 3 show the individual advantages of D5, D6 and D8 domains as DNA barcode candidates. The D5 domain had the smallest intraspecific divergences (0–1.26%), D6 had the largest DNA barcoding gap (10.54%) and the shortest length (192 bp), and D8 had the highest sequencing success rate (77.59%) and the longest indels (241 bp). Therefore, if the use of the three divergent regions was combined, they should complement each other, improving identification efficiency.

**Table 4:** Comparison of the three DNA barcode candidates

*28S*	Intraspecific divergence (%)	Interspecific divergence (%)	DNA barcoding gap (%)	Length (bp)	Indel (bp)
D5	0–1.26	7.46–20.51	6.20 (1.26–7.46)	413–507	94
D6	0–2.27	12.81–23.14	10.54 (2.27–12.81)	192–241	49
D8	0–7.18	13.10–38.75	5.92 (7.18–13.1)	302–543	241

#### Universality in acariformes and parasitiformes

A total of 225 almost complete *28S* gene sequences for Acari were screened from GenBank, which belonged to 225 species of 40 families (186 species of 33 families in Acariformes and 39 species of 7 families in Parasitiformes) (Additional file 4: Table S1). The alignment results of the 225 sequences showed that the three pairs of primers designed for D5, D6 and D8 regions were universal, and base mutations were found in only two primers. The 11th base of the reverse primer (5′-TTG CTA CTA CCA CCA AGA TCT G-3′) of D6 was mutated from “T” to “C” in the Arrenuridae and Acaridae, and the 4th base of the forward primer (5′-GCA KCA GGT CTC CAA GG-3′) of D8 was mutated from “T” to “C” in the Gabuciniidae and Pterolichidae. Corresponding degenerate bases were utilized to replace the two mutation sites. Therefore, the primers for the D5, D6 and D8 regions of the *28S* rRNA gene were also universal for the 225 species of 40 families in both Acariformes (Additional files 5: Figure S4, Additional file 6: Figure S5, Additional file 7: Figure S6) and Parasitiformes (Additional file 8: Figure S7).

## Discussion

Considering that morphological identification of mites is difficult, and DNA identification is hampered by scarce molecular data, the present study provides three main insights. First, to the best of our knowledge, *28S* rDNA was for the first time confirmed as the best potential candidate to be used in the identification and classification of 125 mites belonging to nine medically important mite species of six families. Domains D5, D6 and D8 were suitable DNA barcodes and each had its advantages. D5 had the smallest intraspecific divergences (0–1.26%), which is beneficial for identification of closely related species, while D6 showed the largest DNA barcoding gap (10.54%) between the intraspecific divergences (0–2.27%) and interspecific divergences (12.81–23.14%), which is beneficial for identification of distantly related species. Further, D8 had the highest sequencing success rate (77.59%) and the longest indels (241 bp), which enabled a preliminary discrimination of mite species using agarose-gel electrophoresis of PCR products, and if necessary, further sequencing and alignment could be performed for confirmation. Therefore, the combined use of the three divergent regions should be complementary and improve identification efficiency. Secondly, the universal primers or degenerate primers designed in the present study for D5, D6 and D8 domains had powerful expansibility. They were suitable not only for the identification of the nine medically important mite species, but also for 225 species of 40 families of Acariformes and Parasitiformes in Acari. Thirdly, the methods used in this study are worthy of much wider application and development. By retrieving and aligning molecular data of target genes from GenBank, the universality of the primers could be clearly defined. This provides new insights for molecular identification of thousands of other species besides Acari (which lack molecular data), and will contribute to the initiative of the International Barcode of Life (IBOL) programme.

IBOL was officially launched in 2009. The class Insecta, which comprises the largest number of species, forms the majority of the data in IBOL. The species currently active in IBOL include Lepidoptera (LepBOL), Trichoptera (TrichopteraBOL), Formicidae (FormicidaeBOL), bees (BeeBOL), trypetids (TBI), mosquitoes (MBI), invasive insects (INBIPS) and quarantine insects (QBOL). The medically important mites involved in this study belong to the Acari of arachnids. Although the DNA barcode programme for medically important mites and closely related species has been initiated with high priority, the application of DNA barcoding in mites is limited due to the large number of species, microscopic size, troublesome DNA extraction, and scarcity of specialist researchers. In particular, the lack of molecular data is the most prominent problem for most mite species. Motivated by the situation above, in the present study we successfully screened divergent regions of *28S* rDNA as universal DNA barcodes for mites, based on bioinformatics analysis of the limited mtDNA and rDNA sequences of Acari retrieved from GenBank. This study will therefore have a strong effect on the execution of the DNA barcode programme for medically important mites and closely related species.

DNA barcoding was proposed by Canadian taxonomists Hebert et al. [48] in 2003. *cox*1 mtDNA 648-bp fragment was selected as a universal DNA barcode for global organisms, because it has the advantages of having maternal inheritance, low gene recombination incidence, sufficient mutation, and few indels. Based on the sequence alignment of the nine mite species tested, the present study found that although it could be used for molecular identification of different species, the primers lack universality as they were located at different positions. Even though the universal primers proposed by Herbert et al. [47] (F: 5′-GGT CAA CAA ATC ATA AAG ATA TTG G-3′; R: 5′-TAA ACT TCA GGG TGA CCA AAA AAT C-3′) aligned well with the primers for *C. malaccensis* and *S. canis* [26], in our study they were not universal for the remaining seven mite species belonging to the other four families. This result is not conducive to the feasibility of *cox*1 mtDNA recommended by IBOL for molecular identification of unknown mites. The analysis results for *16S* and *12S* mtDNA were similar to those for *cox*1 mtDNA.

ITS2 rDNA has also been used as a DNA barcode in the molecular identification and classification of some species of mite, such as flour mites, spider mites and gall mites [16, 19, 21], but is not appropriate for the *Sarcoptes* mites. Compared with *cox*1 and *16S* mtDNA gene fragments, ITS2 rDNA could discriminate neither *S. hominis* from *Sarcoptes* spp. infesting animals nor different geographical populations of *S. hominis* as the intraspecific divergences and interspecific divergences almost completely overlapped without a clear DNA barcoding gap [26]. According to the sequence alignment results of the nine medically important mite species from six families, the ITS rDNA region is not a suitable DNA barcode because of the following three reasons. First, the primers for the medically important mites were located at different positions without universality. Secondly, ITS2 rDNA was too variable in sequence composition and sequence length to be aligned well. Thirdly, both the forward primer at the *5.8S* region and the reverse primer at the *28S* region were located at different positions in different mite species. Most importantly, the lack of sequences in some mite species conclusively proved that the ITS rDNA region is not suitable for designing universal primers.

Despite the fact that *18S* and *28S*, as nuclear genes, are rich in eukaryotes, they have not been considered to be suitable for identification and classification at lower taxonomic ranks (species and genus) due to the sequence conservation. However, the present study found that the divergent regions of *28S* rDNA for mite species were more highly variable and rich in mutations, compared with *18S*. In particular, domains D5, D6 and D8 exhibited good identification efficiency, effectively distinguishing species at both higher and lower taxonomic ranks. A significant barcoding gap was found between intraspecific divergences and interspecific divergences in the identification of the nine mite species. More importantly, the universal primers of these three divergent regions can be popularized to 225 species of 40 families in Acariformes and Parasitiformes.

## Conclusions

In conclusion, domains D5, D6 and D8 of *28S* rDNA are ideal areas to be used for molecular identification and classification of mites. The unique structure of the conserved regions and variable regions in the *18S*, *28S* and ITS regions could not only facilitate the design of universal primers, but also make rDNA suitable for classification and identification at different categories. Therefore, at the present stage, *28S* rDNA, as a powerful supplement to *cox*1 mtDNA 648-bp fragment recommended by IBOL, could play a greater role in the molecular identification of mites.

## Supplementary information

**Additional file 1: Figure S1.** Labeled primers of mtDNA gene fragments for mites involved in this study. **a***cox*1; **b***16S*; **c***12S*.

**Additional file 2: Figure S2.** Labeled primers of rDNA regions for mites involved in this study. **a** ITS2; **b***18S*.

**Additional file 3: Figure S3.** Labeled primers of *28S* rDNA for mites involved in this study.

**Additional file 4: Table S1.** Information for *28S* rDNA sequences involved in designing universal primers across Acari.

**Additional file 5: Figure S4.** Universal primers alignment of *28S* rDNA D5 domain in 186 mite species of 33 families across Acariformes.

**Additional file 6: Figure S5.** Universal primers alignment of *28S* rDNA D6 domain in 186 mite species of 33 families across Acariformes.

**Additional file 7: Figure S6.** Universal primers alignment of *28S* rDNA D8 domain in 186 mite species of 33 families across Acariformes.

**Additional file 8: Figure S7.** Universal primers alignment of *28S* rDNA domains D5, D6 and D8 in 39 mite species of 7 families across Parasitiformes. **a** D5; **b** D6; **c** D8.

## Data Availability

Data supporting the conclusions of this article are included in the article and its additional files. The newly generated sequences were submitted to the GenBank database under the accession numbers listed in Table 3.

## Abbreviations

IBOL: International Barcode of Life
*18S*: small subunit ribosomal RNA gene
*28S*: large subunit ribosomal RNA gene
rDNA: ribosomal DNA
*cox*1: cytochrome *c* oxidase subunit 1 gene
ITS: internal transcribed spacer
PCR: polymerase chain reaction
BLAST: Basic Local Alignment Search Tool
ML: maximum likelihood
BI: Bayesian inference
K2P: Kimura 2-parameter
AIC: Akaike information criterion
bs: bootstrap support
pp: posterior probability
LepBOL: Lepidoptera Barcode of Life
TrichopteraBOL: Trichoptera Barcode of Life
Formicidae BOL: Formicidae Barcode of Life
BeeBOL: bees Barcode of Life Initiative
TBI: Trypetid Barcode of Life Initiative
MBI: mosquitoes Barcode of Life Initiative
INBIPS: invasive insects Barcode of Life
QBOL: quarantine insects Barcode of Life
