# Supplementary figures and images for: Divergent domains of 28S ribosomal RNA gene: DNA barcodes for molecular classification and identification of mites
Source: Parasit Vectors. 2020 May 13;13:251. doi: 10.1186/s13071-020-04124-z (PMC7222323; doi:10.1186/s13071-020-04124-z)

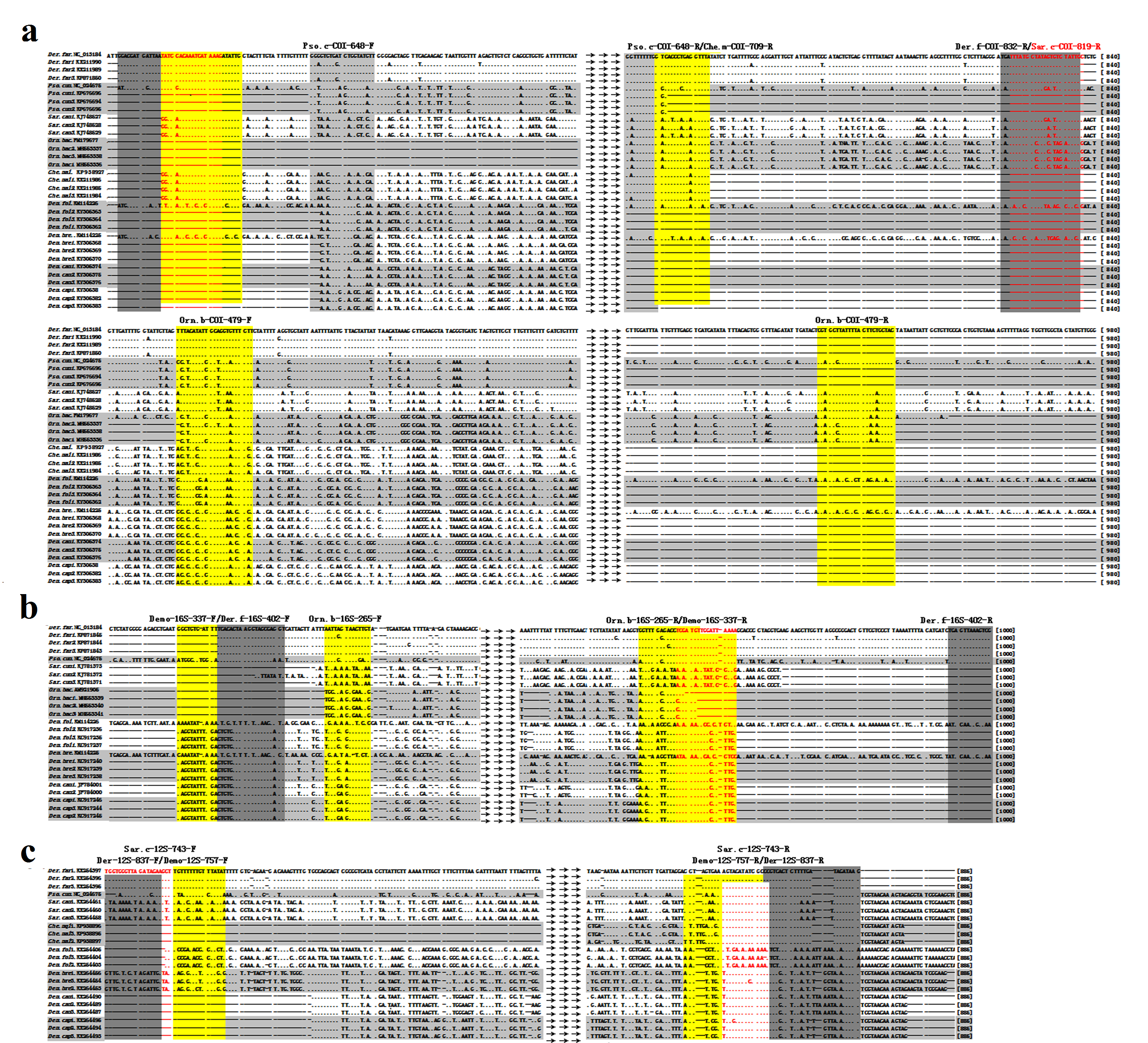

Supplement: Supplementary file 1 — Additional file 1: Figure S1. Labeled primers of mtDNA gene fragments for mites involved in this study. acox1; b16S; c12S. [file 13071_2020_4124_MOESM1_ESM.tif]

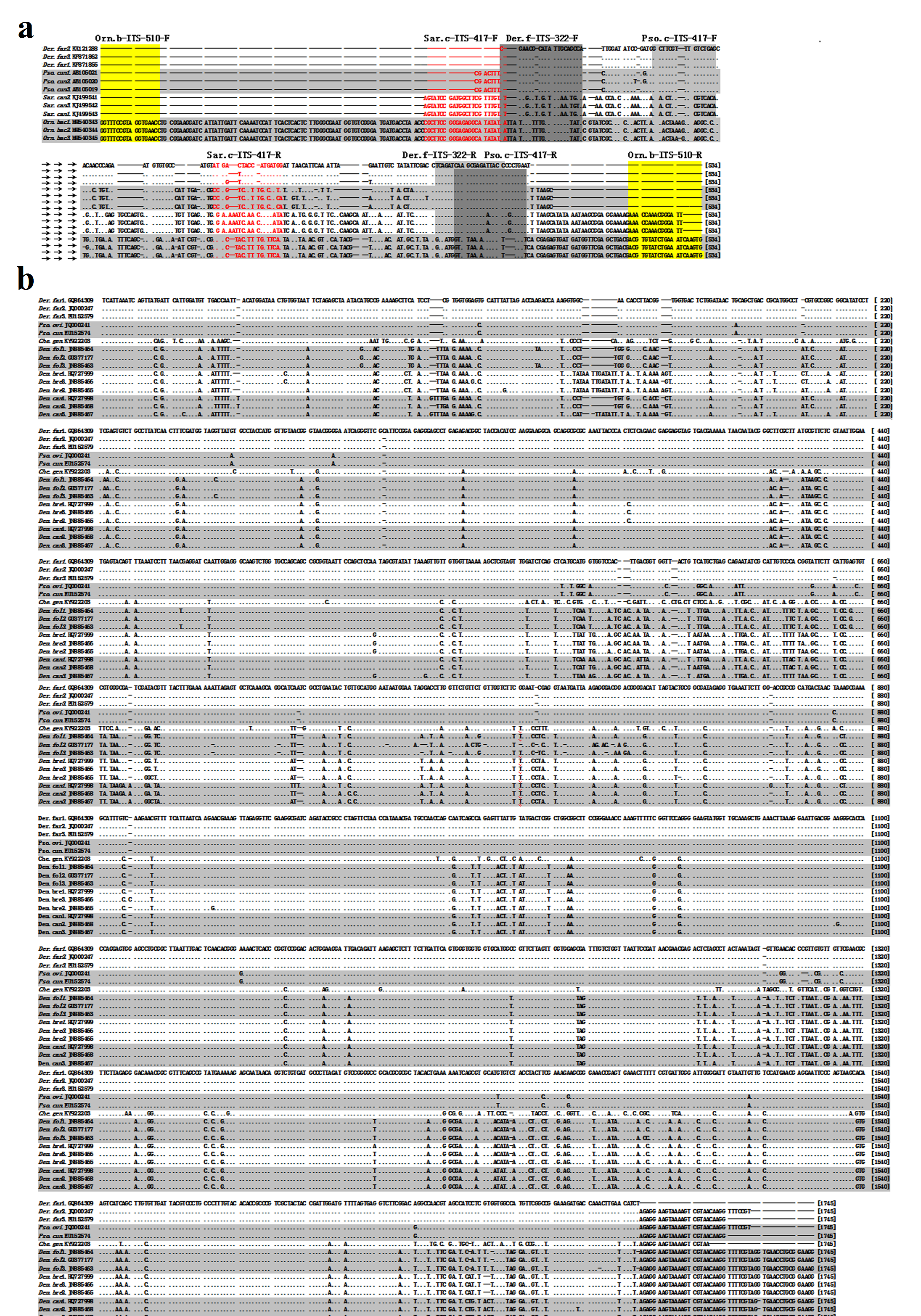

Supplement: Supplementary file 2 — Additional file 2: Figure S2. Labeled primers of rDNA regions for mites involved in this study. a ITS2; b18S. [file 13071_2020_4124_MOESM2_ESM.tif]

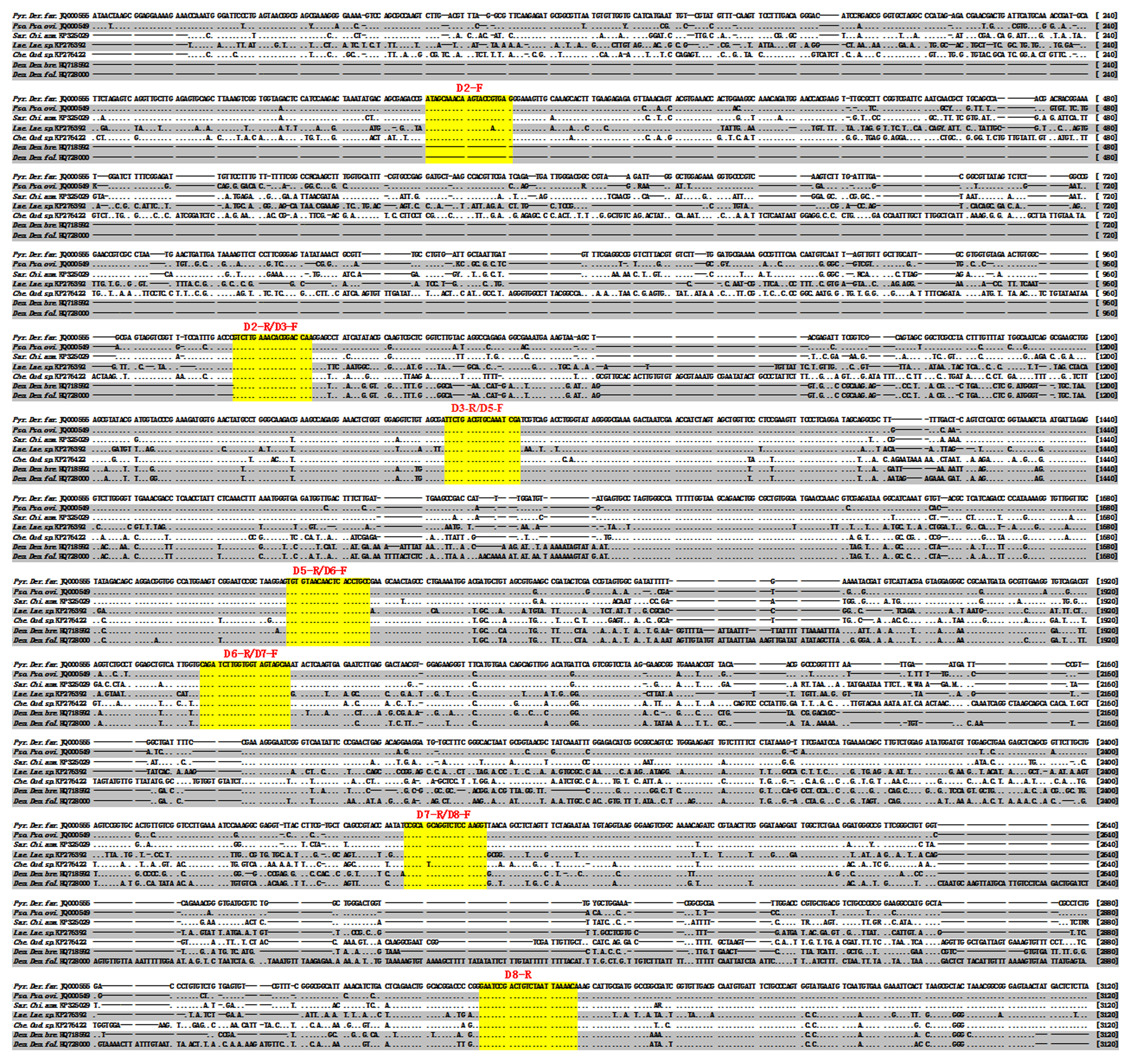

Supplement: Supplementary file 3 — Additional file 3: Figure S3. Labeled primers of 28S rDNA for mites involved in this study. [file 13071_2020_4124_MOESM3_ESM.tif]

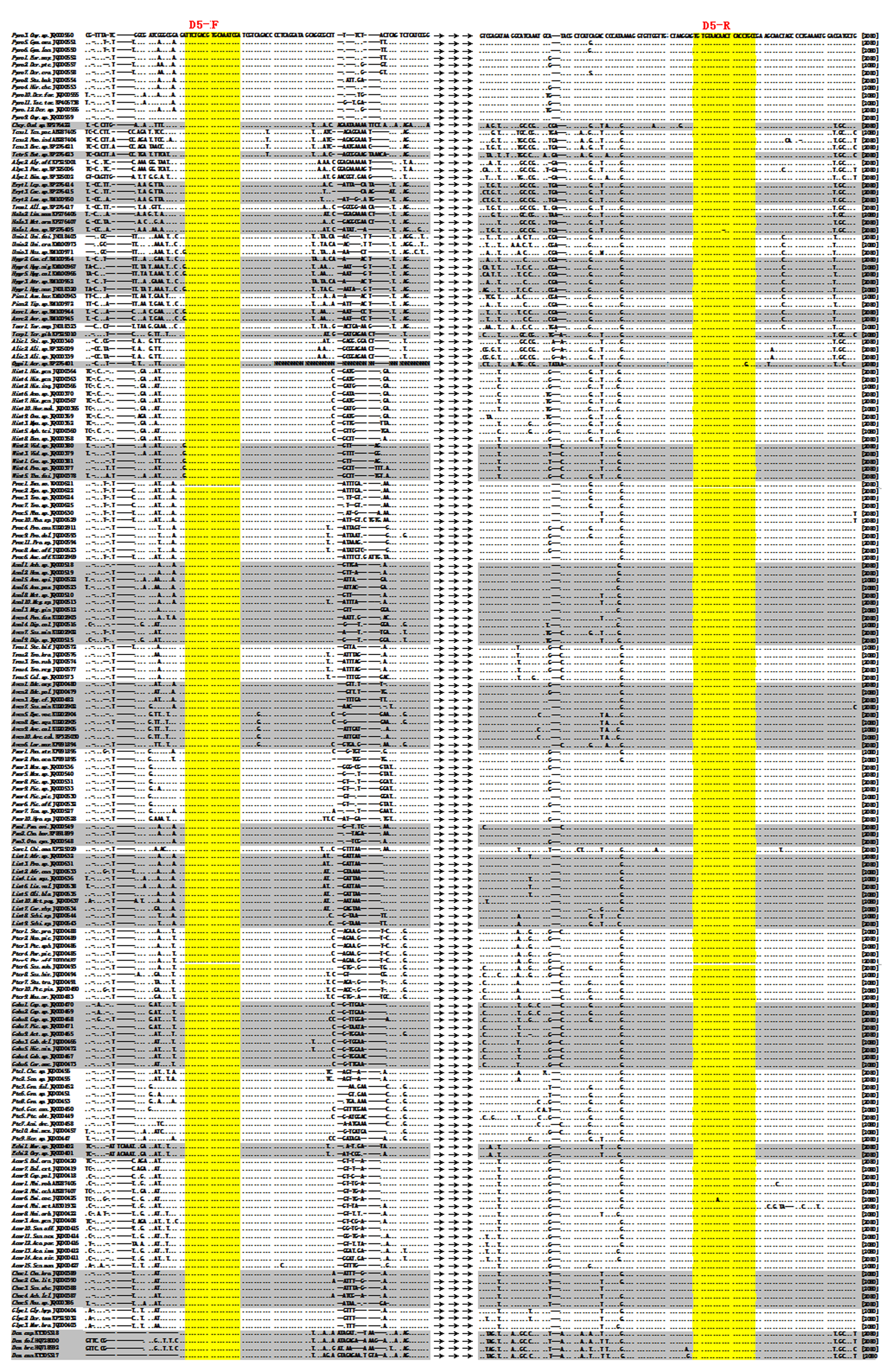

Supplement: Supplementary file 5 — Additional file 5: Figure S4. Universal primers alignment of 28S rDNA D5 domain in 186 mite species of 33 families across Acariformes. [file 13071_2020_4124_MOESM5_ESM.tif]

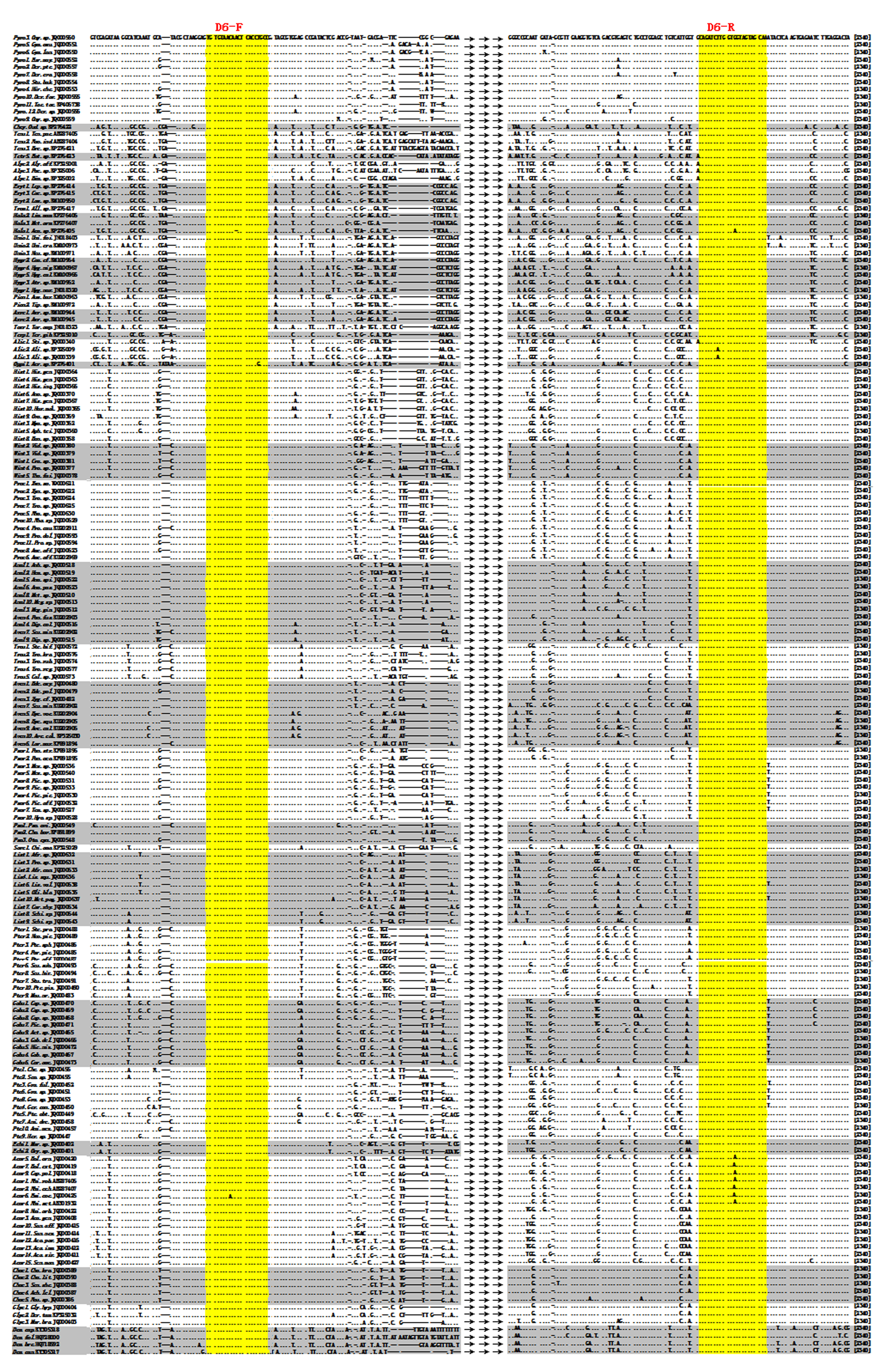

Supplement: Supplementary file 6 — Additional file 6: Figure S5. Universal primers alignment of 28S rDNA D6 domain in 186 mite species of 33 families across Acariformes. [file 13071_2020_4124_MOESM6_ESM.tif]

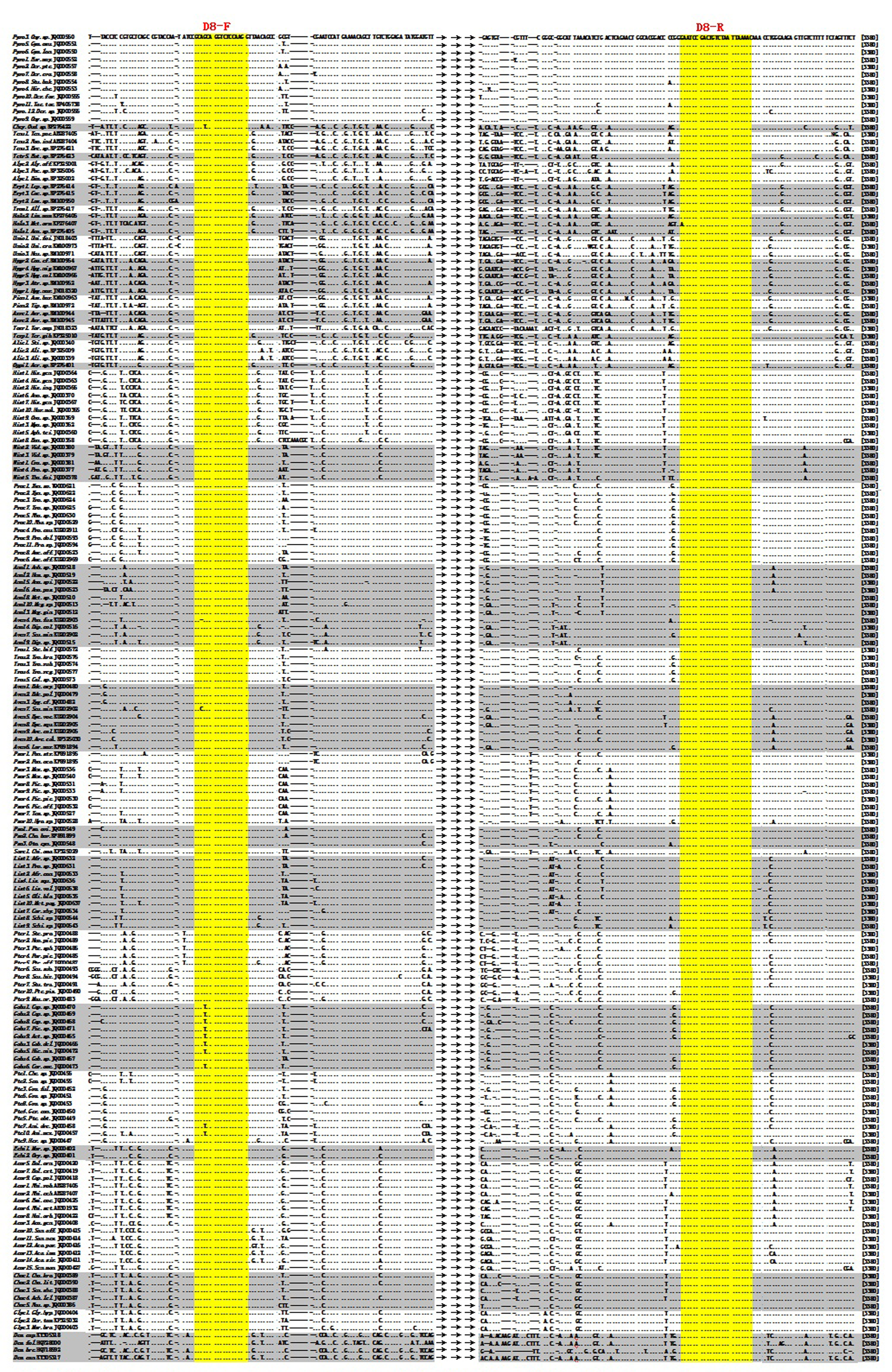

Supplement: Supplementary file 7 — Additional file 7: Figure S6. Universal primers alignment of 28S rDNA D8 domain in 186 mite species of 33 families across Acariformes. [file 13071_2020_4124_MOESM7_ESM.tif]

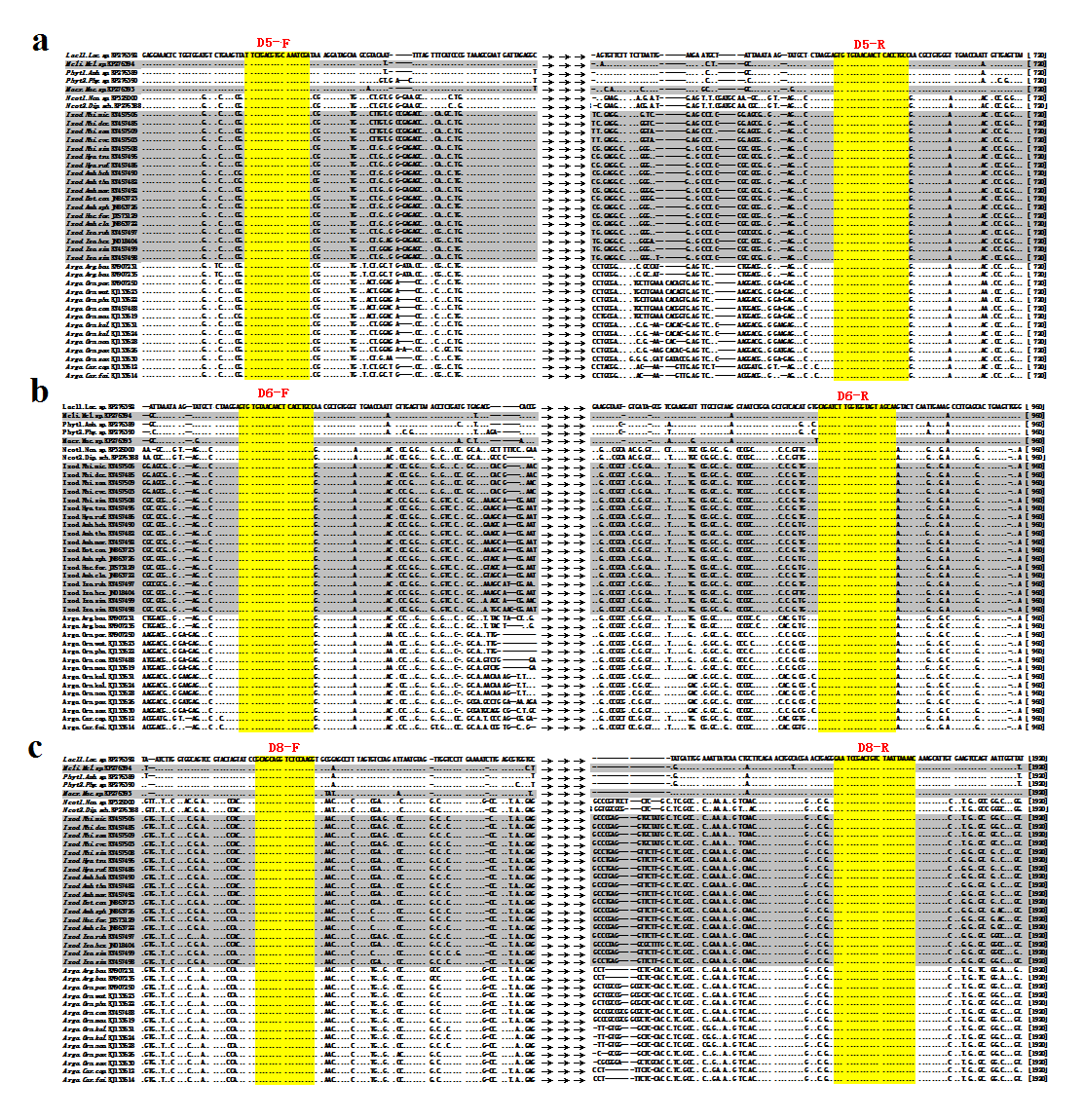

Supplement: Supplementary file 8 — Additional file 8: Figure S7. Universal primers alignment of 28S rDNA domains D5, D6 and D8 in 39 mite species of 7 families across Parasitiformes. a D5; b D6; c D8. [file 13071_2020_4124_MOESM8_ESM.tif]
