# Supplementary material for: Divergent domains of 28S ribosomal RNA gene: DNA barcodes for molecular classification and identification of mites
Source: Parasit Vectors. 2020 May 13;13:251. doi: 10.1186/s13071-020-04124-z (PMC7222323; doi:10.1186/s13071-020-04124-z)
Supplement: Supplementary file 4 — Additional file 4: Table S1. Information for 28S rDNA sequences involved in designing universal primers across Acari. [file 13071_2020_4124_MOESM4_ESM.docx]

**Additional file 4: Table S1** Information of 28S rDNA sequences involved in designing universal primers across Acari

| Family | Species | Acc no. | S Source | Family | Species | Acc no. | Source |
| --- | --- | --- | --- | --- | --- | --- | --- |
| Pyroglyphidae | Onychalges | JQ000560 | USA,2013 | Analgidae | Anhemialges | JQ000518 | USA,2013 |
|  | Gymnoglyphus | JQ000551 | USA,2013 |  | Hemialges | JQ000519 | USA,2013 |
|  | Gymnoglyphus | JQ000550 | USA,2013 |  | Megninia | JQ000512 | USA,2013 |
|  | Euroglyphus | JQ000552 | USA,2013 |  | Diplaegidia | JQ000516 | USA,2013 |
|  | Dermatophagoides1 | JQ000557 | USA,2013 |  | Analges | JQ000522 | USA,2013 |
|  | Dermatophagoides2 | JQ000558 | USA,2013 |  | Analges | JQ000523 | USA,2013 |
|  | Sturnophagoides | JQ000554 | USA,2013 |  | Micralges | JQ000514 | USA,2013 |
|  | Hirstia | JQ000553 | USA,2013 |  | Metanalges | JQ000510 | USA,2013 |
|  | Dermatophagoides3 | JQ000555 | USA,2013 |  | Diplaegidia | JQ000515 | USA,2013 |
|  | Tachornithoglyphus | KP406738 | USA,2015 |  | Megniniella | JQ000513 | USA,2013 |
|  | Dermatophagoides4 | JQ000556 | USA,2013 | Trouessartiidae | Steatacarus | JQ000572 | USA,2013 |
|  | Onychalges | JQ000559 | USA,2013 |  | Trouessartia1 | JQ000576 | USA,2013 |
|  | Dermatophagoides5 | KP406737 | USA,2015 |  | Trouessartia2 | JQ000574 | USA,2013 |
| Cheyletidae | Oudemansicheyla | KP276422 | Brazil,2015 |  | Trouessartia3 | JQ000577 | USA,2013 |
| Tenuipalpidae | Tenuipalpus | AB287405 | Taiwan,2008 |  | Calcealges | JQ000573 | USA,2013 |
|  | Raoiella | AB287404 | Taiwan,2008 | Avenzoariidae | Bdellorhynchus1 | JQ000480 | USA,2013 |
|  | Brevipalpus | KP276421 | Brazil,2015 |  | Bdellorhynchus2 | JQ000479 | USA,2013 |
| Tetranychidae | Eotetranychus | KP276423 | Brazil,2015 |  | Zygochelifer | JQ000482 | USA,2013 |
| Alycidae | Alycus | KP325001 | Brazil,2015 |  | Pandionacarus | KU202903. | USA,2016 |
|  | Pachygnathus | KP325006 | Brazil,2015 |  | Bychovskiata1 | KU202904 | USA,2016 |
|  | Bimichaelia | KP325002 | Brazil,2015 |  | Bychovskiata2 | KU202905 | USA,2016 |
| Erythraeoidea | Leptus | KP276414 | Brazil,2015 |  | Laronyssus | KF891894 | USA,2015 |
|  | Caeculisoma | KP276415 | Brazil,2015 |  | Scutomegninia | KU202902 | USA,2016 |
|  | Lasioerythraeus | KM100950 | Poland,2015 |  | Avenzoaria1 | KU202906 | USA,2016 |
| Trombidiidae | Allothrombium | KP276417 | Brazil,2015 |  | Avenzoaria2 | KP325030 | Brazil,2015 |
| Arrenuridae | Limnohalacarus | KP276406 | Brazil,2015 | Psoroptoididae | Pandalura | KF891896 | USA,2015 |
|  | Metarhombognathus | KP276407 | Brazil,2015 |  | Pandalura | KF891895 | USA,2015 |
|  | Acarothrix | KP276405 | Brazil,2015 |  | Picalgoides1 | JQ000530 | USA,2013 |
|  | Arrenurus | KM100944 | Poland,2015 |  | Picalgoides2 | JQ000532 | USA,2013 |
|  | Arrenurus | KM100945 | Poland,2015 |  | Picalgoides3 | JQ000531 | USA,2013 |
| Unionicolidae | Unionicola | JN018403 | France,2012 |  | Picalgoides4 | JQ000533 | USA,2013 |
|  | Unionicola | KM100973 | Poland,2015 |  | Mesalgoides1 | JQ000536 | USA,2013 |
|  | Neumania | KM100971 | Poland,2015 |  | Mesalgoides2 | JQ000540 | USA,2013 |
| Hygrobatidae | Coaustraliobates | KM100964 | Poland,2015 |  | Hyomesalges | JQ000528 | USA,2013 |
|  | Hygrobates | KM100967 | Poland,2015 |  | Temnalges | JQ000527 | USA,2013 |
|  | Hygrobates | KM100966 | Poland,2015 | Psoroptidae | Psoroptes | JQ000549 | USA,2013 |
|  | Atractides | KM100962 | Poland,2015 |  | Chorioptes | KF891899 | USA,2015 |
|  | Hygrobates | JN018320 | France,2012 |  | Otodectes | JQ000548 | USA,2013 |
| Pionidae | Australotiphys | KM100963 | Poland,2015 | Sarcoptidae | Chirnyssoides | KP325029 | Brazil,2015 |
|  | Tiphys | KM100972 | Poland,2015 | Listrophoridae | Afrolistrophorus1 | JQ000632 | USA,2013 |
| Torrenticolidae | Torrenticola | JN018323 | France,2012 |  | Afrolistrophorus2 | JQ000633 | USA,2013 |
| Terpnacaridae | Terpnacarus | KP325010 | Brazil,2015 |  | Listrophorus1 | JQ000636 | USA,2013 |
| Alicorhagiidae | Stigmalychus | JQ000340 | USA,2013 |  | Listrophorus2 | JQ000638 | USA,2013 |
|  | Alicorhagia | KP325009 | USA,2013 |  | Schizocarpus1 | JQ000644 | USA,2013 |
|  | Alicorhagia | JQ000339 | USA,2013 |  | Schizocarpus2 | JQ000643 | USA,2013 |
| Oppiidae | Aeroppia | KP276401 | Brazil,2015 |  | Prolistrophorus | JQ000631 | USA,2013 |
| Histiostomatidae | Histiostomatidae1 | JQ000364 | USA,2013 |  | Carnilistrophorus | JQ000634 | USA,2013 |
|  | Histiostomatidae2 | JQ000363 | USA,2013 |  | Metalistrophorus | JQ000637 | USA,2013 |
|  | Histiostoma | JQ000366 | USA,2013 |  | Olistrophorus | JQ000635 | USA,2013 |
|  | Histiostomatidae3 | JQ000367 | USA,2013 | Pteronyssidae | Stenopteronyssus | JQ000488 | USA,2013 |
|  | Anoetus | JQ000370. | USA,2013 |  | Neopteronyssus | JQ000489 | USA,2013 |
|  | Ovanoetus | JQ000369 | USA,2013 |  | Pteronyssus | JQ000486 | USA,2013 |
|  | Hormosianoetus | JQ000365 | USA,2013 |  | Pteronyssus | JQ000487 | USA,2013 |
|  | Myanoetus | JQ000362 | USA,2013 |  | Pteronyssoides | JQ000490 | USA,2013 |
|  | Aphodanoetus | JQ000360 | USA,2013 |  | Sturnotrogus | JQ000491 | USA,2013 |
|  | Bonomoia | JQ000358 | USA,2013 |  | Scutulanyssus1 | JQ000493 | USA,2013 |
| Winterschmidtiidae | Crabrovidia | JQ000381 | USA,2013 |  | Scutulanyssus2 | JQ000494 | USA,2013 |
|  | Vidia1 | JQ000380 | USA,2013 |  | Mouchetia | JQ000483. | USA,2013 |
|  | Vidia2 | JQ000379 | USA,2013 |  | Parapteronyssus | JQ000485 | USA,2013 |
|  | Procalvolia | JQ000377 | USA,2013 | Gabuciniidae | Capitolichus1 | JQ000470 | USA,2013 |
|  | Thalassophagacarus | JQ000378 | USA,2013 |  | Capitolichus2 | JQ000469 | USA,2013 |
| Proctophyllodidae | Xynonodectes1 | JQ000621 | USA,2013 |  | Capitolichus3 | JQ000468 | USA,2013 |
|  | Xynonodectes2 | JQ000622 | USA,2013 |  | Gabucinia1 | JQ000467 | USA,2013 |
|  | Trochilodectes | JQ000624 | USA,2013 |  | Gabucinia2 | JQ000466 | USA,2013 |
|  | Proctophyllodes1 | KU202911 | USA,2016 |  | Hieracolichus | JQ000472 | USA,2013 |
|  | Proctophyllodes2 | JQ000593 | USA,2013 |  | Aetacarus | JQ000465 | USA,2013 |
|  | Proctophyllodes3 | JQ000594 | USA,2013 |  | Coraciacarus | JQ000473 | USA,2013 |
|  | Rhamphocaulus | JQ000630 | USA,2013 |  | Piciformobia | JQ000471 | USA,2013 |
|  | Amerodectes1 | KU202969 | USA,2016 | Demodicidae | Demodex | KY305318 | Chian,2018 |
|  | Amerodectes2 | JQ000623 | USA,2013 |  | Demodex | HQ728000 | Chian,2012 |
|  | Trochilodectes | JQ000625 | USA,2013 |  | Demodex | HQ718592 | Chian,2012 |
|  | Rhamphocaulus | JQ000629 | USA,2013 |  | Demodex | KY305317 | Chian,2018 |
| Pterolichidae | Chelomatolichus | JQ000456 | USA,2013 | Echimyopodidae | Marmosopus | JQ000402 | USA,2013 |
|  | Scolaralichus | JQ000455 | USA,2013 |  | Oryzomyopus | JQ000401 | USA,2013 |
|  | Grallobia | JQ000452 | USA,2013 | Acaridae | Rhizoglyphus1 | AB287406 | Taiwan,2008 |
|  | Geranolichus | JQ000450 | USA,2013 |  | Rhizoglyphus2 | AB287407 | Taiwan,2008 |
|  | Pterolichus | JQ000449 | USA,2013 |  | Rhizoglyphus3 | AB301932 | Taiwan,2008 |
|  | Grallobia | JQ000451 | USA,2013 |  | Acaridae | JQ000408 | USA,2013 |
|  | Aniibius | JQ000458 | USA,2013 |  | Boletoglyphus1 | JQ000420 | USA,2013 |
|  | Grallolichus | JQ000453 | USA,2013 |  | Boletoglyphus2 | JQ000419 | USA,2013 |
|  | Herodialges | JQ000447 | USA,2013 |  | Ewingia | JQ000425 | USA,2013 |
|  | Aniacarus | JQ000457 | USA,2013 |  | Naiadacarus | JQ000422 | USA,2013 |
| Chaetodactylidae | Chaetodactylus1 | JQ000389 | USA,2013 |  | Capillaroglyphus | JQ000418 | USA,2013 |
|  | Chaetodactylus2 | JQ000390 | USA,2013 |  | Sancassania1 | JQ000415 | USA,2013 |
|  | Sennertia | JQ000388 | USA,2013 |  | Sancassania2 | JQ000414 | USA,2013 |
|  | Achaetodactylus | JQ000387 | USA,2013 |  | Sennertionyx | JQ000427 | USA,2013 |
|  | Roubikia | JQ000386 | USA,2013 |  | Acotyledon | JQ000416 | USA,2013 |
| Glycyphagidae | Glycyphagus | JQ000404 | USA,2013 |  | Acarus | JQ000412 | USA,2013 |
|  | Dermacarus | KP325032 | Brazil,2015 |  | Acarus | JQ000411 | USA,2013 |
|  | Marsupialichus | JQ000403 | USA,2013 |  |  |  |  |
| Laelapidae | Laelapidae | KP276392 | Brazil,2015 | Ixodidae | Rhi.microplus | KY457506 | South Africa,2018 |
| Melicharidae | Melicharidae sp | KP276394.1 | Brazil,2015 |  | Rhi.decoloratus | KY457485 | South Africa,2018 |
| Phytoseiidae | Amblyseius | KP276389 | Brazil,2015 |  | Rhi.zambeziensis | KY457509 | South Africa,2018 |
|  | Phytoseius | KP276390 | Brazil,2015 |  | Rhi.evertsi. | KY457503 | South Africa,2018 |
| Macrochelidae | Macrocheles | KP276393 | Brazil,2015 |  | Rhi.simus | KY457508 | South Africa,2018 |
| Neothyridae | Neothyridae | KP325000 | Brazil,2015 |  | Hyalomma.truncatum. | KY457496 | South Africa,2018 |
|  | Diplothyrus | KP276388 | Brazil,2015 |  | Hyalomma.rufipes | KY457486 | South Africa,2018 |
| Argasidae | Argas.boueti | KR907231 | South Africa,2017 |  | Amb.hebraeum | KY457490 | South Africa,2018 |
|  | Argas.boueti | KR907235 | South Africa,2017 |  | Amb.tholloni | KY457482 | South Africa,2018 |
|  | Orn.porcinus | KR907250 | South Africa,2017 |  | Amb.marmoreum. | KY457492 | South Africa,2018 |
|  | Orn.waterbergensis | KJ133623 | South Africa,2018 |  | Bot.concolor | JN863723 | Australia,2012 |
|  | Orn.phacochoerus | KJ133622 | South Africa,2018 |  | Amb.sphenodonti | JN863726 | Australia,2012 |
|  | Orn.compactus | KY457488 | South Africa,2018 |  | Hae.formosensis | JX573129 | Australia,2013 |
|  | Orn.moubata | KJ133619 | South Africa,2018 |  | Amb.elaphense. | JN863722 | Australia,2012 |
|  | Orn.kalahariensis | KJ133631 | South Africa,2018 |  | Ixodes.rubicundus | KY457497 | South Africa,2018 |
|  | Orn.kalahariensis | KJ133624 | South Africa,2018 |  | Ixodes.hexagonus. | JN018404 | France,2012 |
|  | Orn.noorsveldensis | KJ133628 | South Africa,2018 |  | Ixodes.simplex | KY457499 | South Africa,2018 |
|  | Orn.pavimentosus | KJ133626 | South Africa,2018 |  | Ixodes.simplex | KY457498 | South Africa,2018 |
|  | Orn.savignyi | KJ133630 | South Africa,2018 |  |  |  |  |
|  | Carios.capensis | KJ133612 | South Africa,2018 |  |  |  |  |
|  | Carios.faini | KJ133614 | South Africa,2018 |  |  |  |  |

Acc no.: accession number.
